# Inhibition of interleukin-6 decreases atrogene expression and ameliorates tail suspension-induced skeletal muscle atrophy

**DOI:** 10.1371/journal.pone.0191318

**Published:** 2018-01-19

**Authors:** Mitsutaka Yakabe, Sumito Ogawa, Hidetaka Ota, Katsuya Iijima, Masato Eto, Yasuyoshi Ouchi, Masahiro Akishita

**Affiliations:** 1 Department of Geriatric Medicine, Graduate School of Medicine, The University of Tokyo, Bunkyo-ku, Tokyo, Japan; 2 Federation of National Public Service Personnel Mutual Aid Associations, Toranomon Hospital, Minato-ku, Tokyo, Japan; University of Minnesota Medical Center, UNITED STATES

## Abstract

**Background:**

Interleukin-6 (IL-6) is an inflammatory cytokine. Whether systemic IL-6 affects atrogene expression and disuse-induced skeletal muscle atrophy is unclear.

**Methods:**

Tail-suspended mice were used as a disuse-induced muscle atrophy model. We administered anti-mouse IL-6 receptor antibody, beta-hydroxy-beta-methylbutyrate (HMB) and vitamin D to the mice and examined the effects on atrogene expression and muscle atrophy.

**Results:**

Serum IL-6 levels were elevated in the mice. Inhibition of IL-6 receptor suppressed muscle RING finger 1 (MuRF1) expression and prevented muscle atrophy. HMB and vitamin D inhibited the serum IL-6 surge, downregulated the expression of MuRF1 and atrogin-1 in the soleus muscle, and ameliorated atrophy in the mice.

**Conclusion:**

Systemic IL-6 affects MuRF1 expression and disuse-induced muscle atrophy.

## Introduction

Skeletal muscle atrophy occurs in many chronic diseases and under conditions of disuse, such as denervation and immobilization [1]. Particularly among the elderly, atrophy can cause sarcopenia and lead to adverse outcomes such as physical disability, poor quality of life and high mortality [2].

Several mechanisms are thought to be involved in disuse-induced muscle atrophy. One mechanism is increased proteolysis. In atrophying muscle, protein degradation is increased through the activation of the ubiquitin-proteasome pathway. The two atrogenes muscle RING finger 1 (MuRF1) and atrogin-1 are well-studied ubiquitin ligases that are thought to promote atrophy. These genes have been identified as molecular mediators of muscle atrophy [3, 4] and are upregulated during atrophy due to conditions such as denervation and immobilization, and mice deficient in either MuRF1 or atrogin-1 have been found to be resistant to atrophy [3]. Forkhead box O (FOXO) transcription factors are known to be recruited to the promoters of MuRF1 and atrogin-1 to activate the transcription of these genes [5, 6]. Unloading-associated muscle atrophy has been supposed to be driven by signals in the immobilized region, rather than systemic factors [7].

Beta-hydroxy-beta-methylbutyrate (HMB) is a metabolite of leucine, one of the ketogenic amino acids. Several studies have focused on the effects of HMB on atrogene expression. HMB prevented dexamethasone-induced muscle wasting by inhibiting the MuRF1 and atrogin-1 expression in rat myotubes *in vitro* [8]. HMB also attenuated dexamethasone-induced muscle atrophy by regulating FOXO transcription factor and subsequent MuRF1 expression in rats [9]. In aged male rats, HMB reduced the expression of MuRF1 [10]. HMB has also been suggested to repress the expression of IL-6 [11, 12]. 1,25-dihydroxyvitamin D (1,25(OH)_2_D_3_) is the hormonal form of vitamin D [13], and most of their function is mediated by a nuclear receptor Vitamin D receptor (VDR). 1,25(OH)_2_D_3_ and other forms of vitamin D have been shown to repress IL-6 *in vitro* and *in vivo* [14, 15]. One study has reported that 1,25(OH)_2_D_3_ downregulated MuRF1 and atrogin-1 expression in human myotubes [16]. In this way, HMB and 1,25(OH)_2_D_3_ have been supposed to possess both anti-atrophic effects and IL-6-repressing effects, but the relationship between these effects is unclear.

IL-6 is a pleiotropic cytokine that acts as both a myokine and an inflammatory cytokine [17]. IL-6 is produced by various cells, including monocytes, fibroblasts, vascular endothelial cells and skeletal muscles [18, 19]. IL-6 is released into the systemic circulation from muscles during acute exercise-mediated skeletal muscle contraction [20]. It activates the proliferation of cells in skeletal muscles, including satellite cells [21, 22]. In cultured C2C12 myotubes, knockdown of IL-6 reduced the expression of myogenic factors such as myogenin and α-actin [23]. In genomic IL-6 knockout mice, overloading of muscles failed to induce the expression of the myogenic marker MyoD [24] or to promote satellite cell proliferation [25]. However, some studies have implicated IL-6 in muscle atrophy. Chronic IL-6 administration directly to skeletal muscles induced atrophy [26]. IL-6-transgenic mice exhibited muscle atrophy, which was inhibited by MR16-1, an anti-mouse IL-6 receptor (IL-6R) antibody [27]. In humans, a longitudinal study in the elderly showed that high serum IL-6 level were associated with muscle loss [28]. According to the concept of “inflamm-aging (inflammation +aging)”, inflammatory cytokines, including IL-6, may be involved in age-related diseases, such as atherosclerosis, dementia, type 2 diabetes and osteoporosis [29, 30], and inflamm-aging might also be involved in sarcopenia [31].

Studies using unloading-induced atrophy model have shown that the expression of IL-6 is elevated in the immobilized muscles or skins [32, 33]. However, to the best of our knowledge, no study has examined the effects of systemic IL-6 on disuse-induced muscle atrophy or atrogenes. Furthermore, few studies have examined whether vitamin D and HMB ameliorate disuse-induced muscle atrophy, or whether the effect is via IL-6-related pathways. Therefore, we hypothesized that the inhibition of systemic IL-6 in a disuse-induced muscle atrophy model might be a strategy to repress atrogene expression and to counteract the atrophy. We also hypothesized that anti-atrophic effects of vitamin D and HMB work via IL-6-related pathways. In the present study, we adopted a tail-suspended mouse model, which has been widely used to simulate disuse-induced muscle atrophy [3, 34], and focused on MuRF1 and atrogin-1. After tail suspension was initiated, the maximal reduction in muscle mass was observed by day 14 [35]. Compared with that in control mice, the expression of MuRF1 and atrogin-1 was significantly upregulated by day 3 in the mice subjected to tail suspension; then, the expression of these factors peaked and became similar to control levels by day 14 [36, 37]. We also examined the effects of MR16-1, HMB and vitamin D on atrogene expression and muscle atrophy in the mice. Our data suggested a role of systemic IL-6 in ameliorating muscle atrophy.

## Methods

### Materials

HMB (calcium salt) was purchased from Alfa Aesar (MA, USA). 1,25(OH)_2_D_3_ was purchased from Sigma-Aldrich Japan (Tokyo, Japan). MR16-1, a monoclonal mouse IL-6R-blocking antibody, was donated by Chugai Pharmaceutical Company (Tokyo, Japan).

### Mice

Male C57BL/6J mice were purchased from Nippon CLEA (Tokyo, Japan). The animals were individually housed in similarly designed cages and maintained in a controlled environment (temperature of 24±1°C, humidity of 55 ± 5%) with a 12:12 h light;dark cycle. The mice received standard chow and water ad libitum. After the mice were acclimatized for two weeks, the experiments commenced.

The experimental protocol was approved by the Ethics Committee for Animal Research at the University of Tokyo (permit number: 13-P-66). Animal health was monitored in accordance with the recommendations of the Guide for the Care and Use of Laboratory Animals (the Japanese Society for Laboratory Animal Resources). A humane endpoint was determined according to the criteria of the Japanese Society for Laboratory Animal Resources. At the end of the planned experiments, the mice were immediately euthanized with carbon dioxide. All efforts were made to minimize suffering. No animals died before meeting the criteria for euthanasia.

### Comparison of aged and young mice

The mice were maintained for approximately two years and monitored at least three times a week. One mouse was excluded because it had a tumor and became severely cachexic with age. Finally, five mice reached 104 weeks of age and were then sacrificed. The soleus muscles were weighed, and serum samples were evaluated using IL-6-specific ELISA as described below. As a controls, 15-week-old mice were sacrificed (n = 4).

### Tail suspension

Mice were subjected to tail suspension, which induced muscle atrophy in their hindlimbs. The tail-suspension protocol was as follows. The entire tail of the mouse was covered and protected with medical adhesive tape. A harness made of the tape was attached to the proximal half of the tail, and the mouse was suspended so that the feet of the hindlimbs could not contact the cage floor. The tail was kept horizontal to the floor. The distal end of the harness was attached to a paperclip, which was then attached to a swivel on a cross-bar. The cross-bar was positioned approximately 15 cm above the cage floor. The tail-suspended mice were able to move freely on their forelimbs in the cage and had free access to food and water ad libitum.

Twelve-week-old mice were subjected to two weeks of tail suspension. The mice were sacrificed, and the serum samples and soleus muscles were then collected. The serum IL-6 level was determined using ELISA as described below. In another experiment, twelve-week old mice were subjected to seven days of tail suspension. The mice were sacrificed, and RNA was extracted from the following tissues: whole bone marrow from femurs, soleus muscles, livers, spleens, and small intestines. These mice were compared with control mice of the same age.

During the experiment, the mice were monitored each day, and none of the mice were found to exhibit health or behavioral problems.

### Administration of 1,25(OH)_2_D_3_ or HMB

Eleven-week-old mice were divided into four groups (each n = 4): control, tail suspension, tail suspension+1,25(OH)_2_D_3_, and tail suspension+HMB. Both 1,25(OH)_2_D_3_ and HMB were administered daily by gavage. The former was dissolved in normal corn oil and administered at 0.1μg/kg/day, and the latter was dissolved in distilled water and administered at 340 mg/kg/day similar to previous studies [38, 39]. The control mice received both corn oil and water. Administration was commenced one week before the mice were subjected to tail suspension and was continued during the tail suspension period. To measure muscle weight and cross-sectional area (CSA), the mice were subjected to tail suspension for two weeks and then sacrificed. The muscles were collected and embedded in paraffin blocks. Subsequently, 5-μm sections were cut from the blocks using a Leica microtome (Leica Instruments GmbH, Hubloch, Germany) and transferred to adhesive-coated slides. Hematoxylin-eosin (HE) staining was performed, and the average CSA of the muscles was calculated using ImageJ version 1.44 (National Institutes of Health, USA).

For soleus muscle RNA analysis, other groups of mice were treated with the agents, subjected to tail suspension for three days and then sacrificed.

### RNA analysis

The collected samples (soleus muscles and whole bone marrow) were immersed in RNAlater (Qiagen, CA, USA) to stabilize the RNA in tissues. RNA extraction and DNase I treatment were performed using an RNeasy Fibrous Tissue Mini Kit (Qiagen).

cDNA was synthesized from the RNA samples using the Omniscript RT Kit (Qiagen) and oligo (dT) primers (Invitrogen, CA, USA). The cDNA was mixed with SYBR Green Master Mix (Applied Biosystems, CA, USA) and specific primers for each gene (total amount: 20 μl) and then subjected to quantitative real-time PCR using a 7300 Real-Time PCR System (Applied Biosystems). The amount of amplified cDNA was determined based on the fluorescence of SYBR Green. A dissociation curve was generated to examine the specificity of the PCR reaction. The cycle threshold (Ct) for each gene was normalized to that for GAPDH, which was used as the internal control. All real-time PCR experiments were conducted in triplicate. The primer sequences are shown below.

GAPDH: 5’-AGGTCGGTGTGAACGGATTTG -3’(forward) and 5’-TGTAGACCAGTAGTTGAGGTCA -3’(reverse);

IL-6: 5’-TAGTCCTTCCTACCCCAATTTCC- 3’(forward) and 5’-TTGGTCCTTAGCCACTCCTTC-3’(reverse);

MuRF1: 5’-TGCCTGGAGATGTTTACCAAGC -3’(forward) and 5’-AAACGACCTCCAGACATGGACA -3’(reverse); and

atrogin-1: 5’-AAGGCTGTTGGAGCTGATAG CA -3’(forward) and 5’-CACCCACATGTTAATGTTGCCC-3’(reverse).

### MR16-1 administration protocol

**Fig 1:**
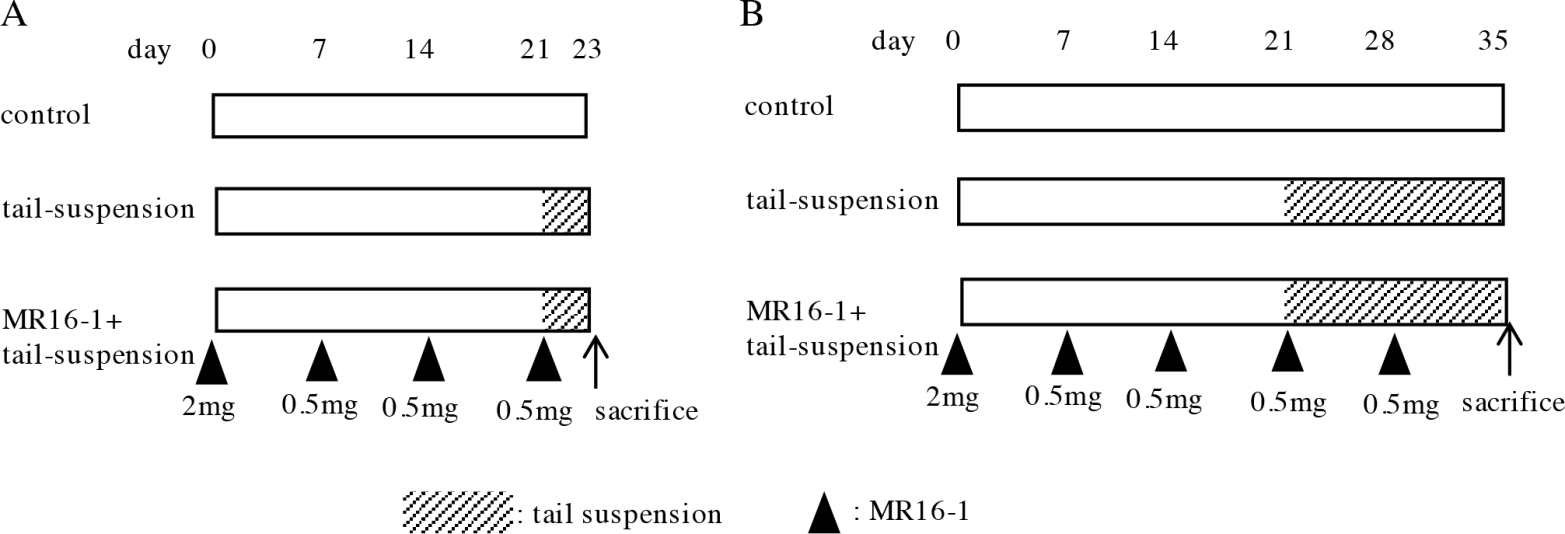
MR16-1 administration protocol. Eleven-week-old C57BL/6J male mice were administered MR16-1 via intraperitoneal injection and subjected to tail suspension. The mice were subjected to two days of tail suspension to examine atrogene expression in the soleus muscles (A, n = 7–8), and to two weeks of tail suspension to measure soleus muscle weight and CSA (B, n = 5). The arrowheads indicate MR16-1 administration and its amount.

Eleven-week-old C57BL/6J mice were administered MR16-1 via intraperitoneal injection and subjected to tail suspension. The administration protocol, which was recommended by Chugai Pharmaceutical Company, is shown in Fig 1. To examine the expression MuRF1 and atrogin-1 in the soleus muscles, the mice were subjected to two days of tail suspension (Fig 1A). To measure soleus muscle weight and CSA, another group of mice were subjected to two weeks of tail suspension (Fig 1B).

### Mouse IL-6 ELISA

Mice were divided into five groups: control, tail suspension, tail suspension+1,25(OH)_2_D_3_, tail suspension+HMB, and tail suspension+1,25(OH)_2_D_3_+HMB (each n = 4). After one week of pretreatment, the mice were subjected to two weeks of tail suspension. The serum IL-6 level was determined using a mouse IL-6 ELISA Kit (Ray Biotech, GA, USA) according to the manufacturer’s protocol.

### Statistical analysis

Data are expressed as the mean ±SEM. Comparisons between two groups were performed using Student’s t-test. Comparisons among multiple groups were performed using ANOVA. A value of P<0.05 was considered significant.

## Results

### Characteristics of tail-suspended mice and aged mice

**Fig 2:**
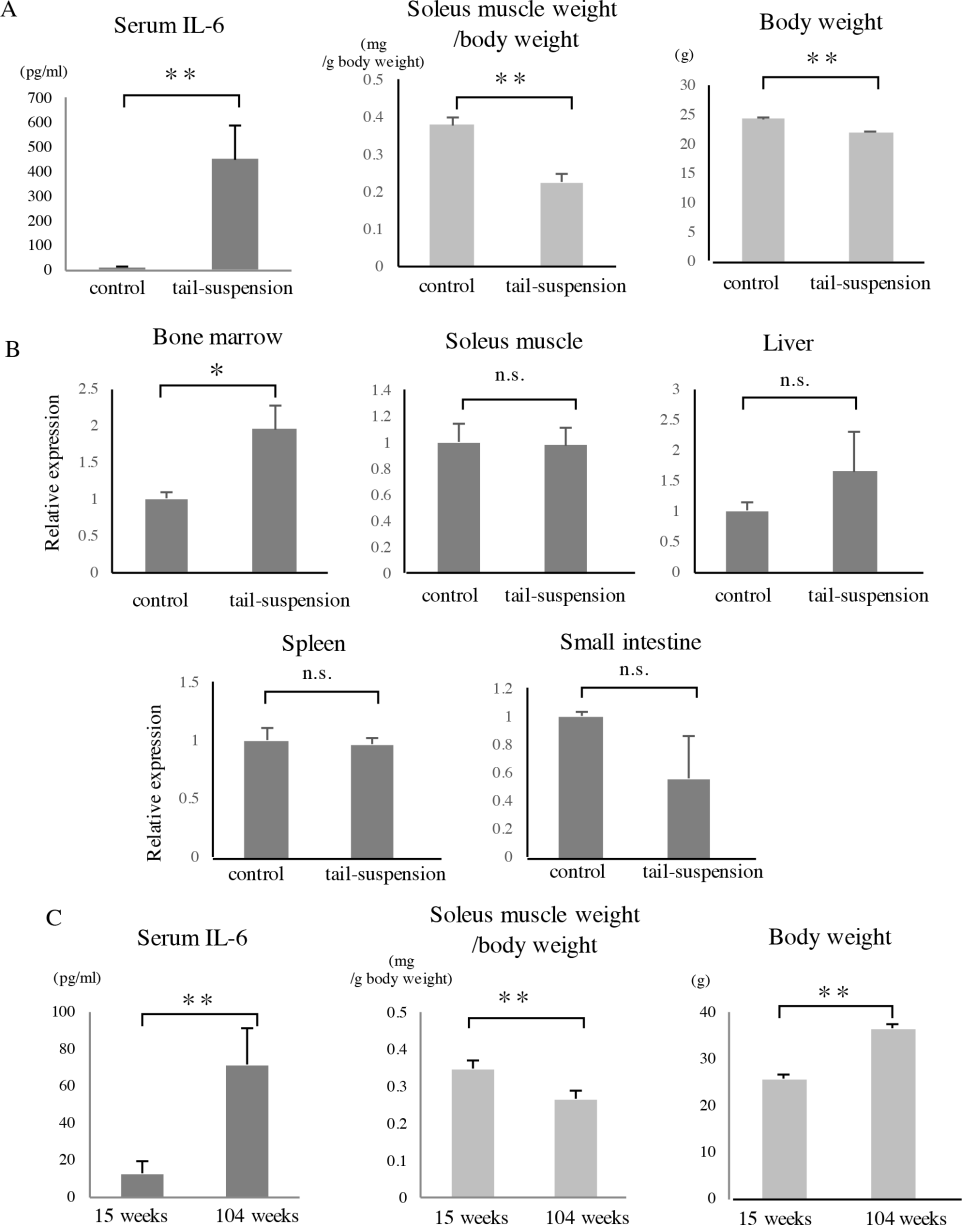
Characteristics of tail-suspended mice and aged mice. (A) Twelve-week-old C57BL/6J male mice were subjected to two weeks of tail suspension. Mice were sacrificed, and then serum samples and soleus muscles were collected. Serum IL-6 level was determined using ELISA. Soleus muscle weight/body weight ratio and the body weight of mice are shown (n = 4). (B) Twelve-week-old mice were subjected to seven days of tail suspension, and then RNA was extracted from the following tissues: whole bone marrow from femurs (n = 4), soleus muscles (n = 3), livers (n = 6), spleens (n = 3), and small intestines (n = 3). Quantitative real-time PCR was performed, and the relative IL-6 expression in each tissue is shown. (C) C57BL/6J male mice aged 15 weeks and 104 weeks were sacrificed, and then serum samples and soleus muscles were collected (n = 4). *p<0.05, **p<0.01, n.s.: not significant.

Mice that were subjected to tail suspension for two weeks exhibited significantly increased serum IL-6 levels (Fig 2A). Tail suspension also induced soleus muscle atrophy, resulting in a decrease in the soleus muscle weight/body weight ratio. To determine the tissue of origin of increased IL-6 in the serum, we subjected another group of mice to seven days of tail suspension and extracted RNA from the following candidate tissues: whole bone marrow from femurs, soleus muscles, livers, spleens, and small intestines. IL-6 expression levels in the bone marrow of these mice were higher than those of the control mice (Fig 2B). In the soleus muscles, no increase in the IL-6 expression was observed. No significant differences were observed in the other tissues.

We also examined the serum IL-6 levels and soleus muscle weight in young and aged mice. The serum IL-6 level was higher in the 104-week-old mice than that in the 15-week-old mice (Fig 2C). The aged mice were heavier than their younger counterparts, and the soleus muscle weight/body weight ratio was lower in the aged mice than that in the young mice.

### Effects of IL-6 inhibition on atrogene expression and muscle atrophy

**Fig 3:**
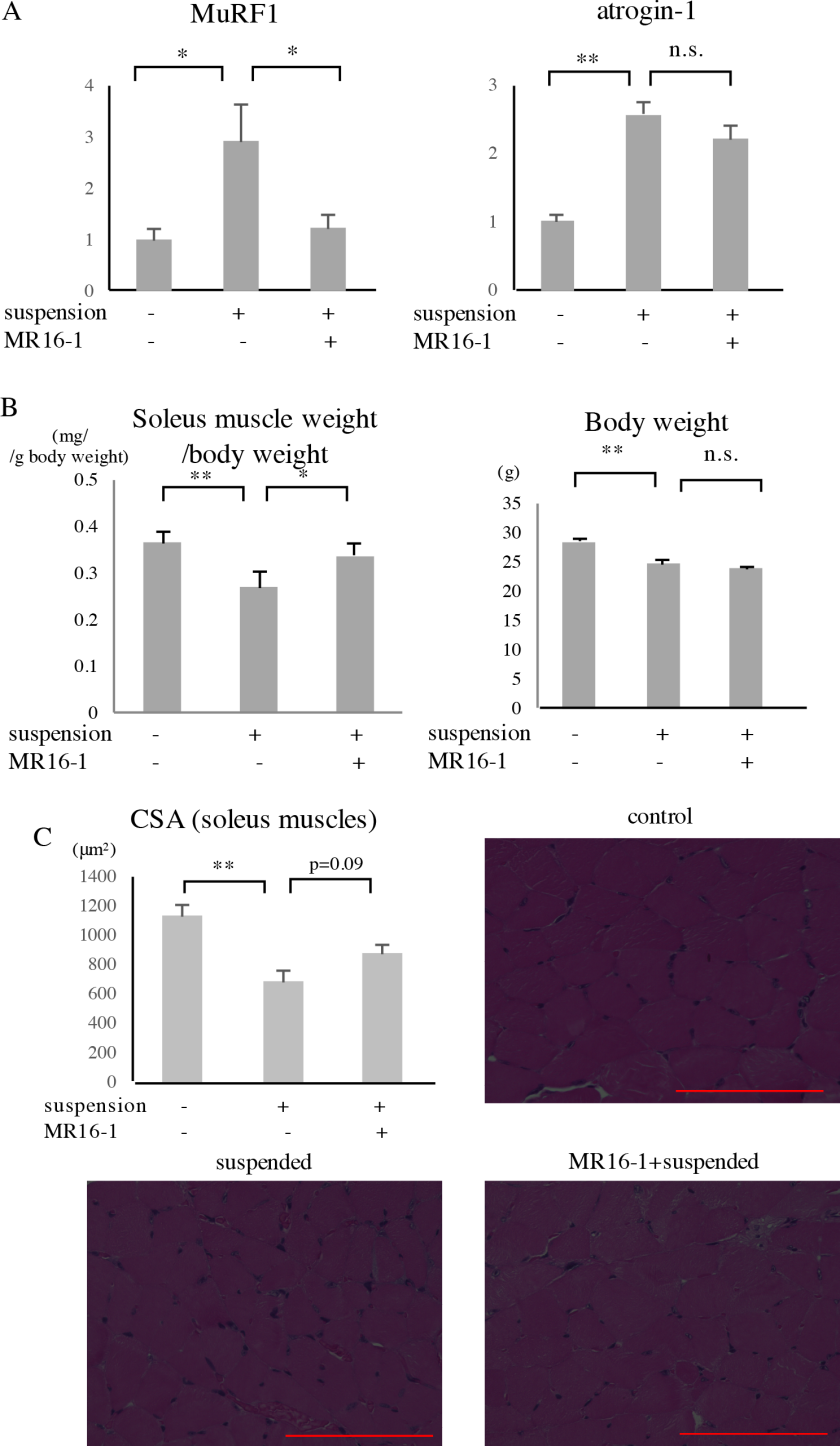
Effects of MR16-1 on atrogene expression and muscle atrophy. Eleven-week-old C57BL/6J male mice were administered with MR16-1 via intraperitoneal injection and subjected to tail suspension according to the protocol in Fig 1. (A) After two days of tail suspension, soleus muscles were collected, and then the expression of MuRF1 and atrogin-1 was measured by real-time PCR. (n = 7–8). (B, C) After two weeks of tail suspension, mice were sacrificed, and soleus muscles were collected. Soleus muscle weight/body weight (B) and CSA of the soleus muscles (C) are shown (n = 5). In Fig 3C, representative microscopic images of the soleus muscles (HE staining) are shown. CSA of each fiber was calculated using ImageJ, and the values were averaged for each muscle. Red bars correspond to 100μm. *p<0.05, **p<0.01, n.s.: not significant.

To examine the effect of IL-6 inhibition on tail suspension-induced muscle atrophy, we administered mice with MR16-1, a monoclonal mouse IL-6R-blocking antibody, according to the protocol shown in Fig 1. The expression of MuRF1 and atrogin-1 in the soleus muscles was significantly increased after two days of tail suspension (Fig 3A). MR16-1 significantly inhibited MuRF1 expression and did not repress atrogin-1 expression.

Another group of mice were subjected to two weeks of tail suspension. MR16-1 significantly ameliorated the reduction in the soleus muscle weight/body weight ratio (Fig 3B). No significant difference in body weight was observed between the tail suspension group and the tail suspension+MR16-1 group. Although the difference was not significant (p = 0.09), MR16-1 also restored the CSA of the soleus muscles (Fig 3C).

### HMB and vitamin D ameliorated muscle atrophy induced by tail suspension

**Fig 4:**
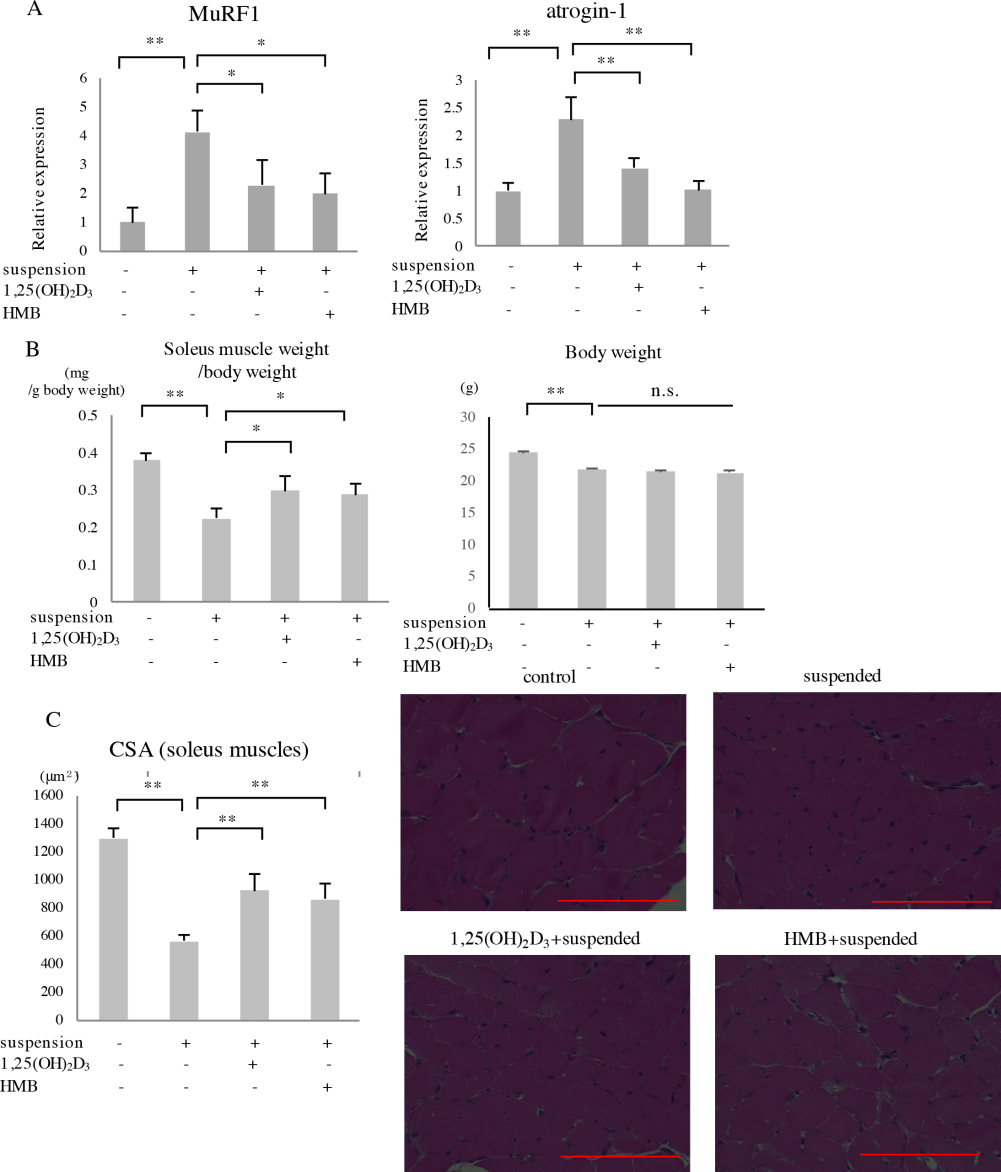
Effects of 1,25(OH)_2_D_3_ and HMB on tail suspension-induced atrogene expression and muscle atrophy. (A) Twelve-week-old C57BL/6J male mice were administered 1,25(OH)_2_D_3_ or HMB by gavage and subjected to three days of tail suspension. MuRF1 and atrogin-1 expression levels were measured by real-time PCR. (B) Twelve-week-old mice were administered 1,25(OH)_2_D_3_ or HMB and subjected to two weeks of tail suspension. Soleus muscle weight/body weight ratio is shown. (C) Values of soleus muscle CSAs measured using ImageJ are shown. Representative images of soleus muscles (HE staining) are shown. Red bars correspond to 100μm. Each n = 4. *p<0.05, **p<0.01, n.s.: not significant.

We determined whether 1,25(OH)_2_D_3_ and HMB could affect atrogene expression and ameliorate muscle atrophy. Both 1,25(OH)_2_D_3_ or HMB inhibited the increase in MuRF1 and atrogin-1 expression (Fig 4A). Two weeks of tail suspension induced muscle atrophy, and both 1,25(OH)_2_D_3_ and HMB ameliorated this atrophy (Fig 4B). The CSA of the soleus muscle was reduced by tail suspension, and both 1,25(OH)_2_D_3_ and HMB partially restored the CSA values (Fig 4C).

### Effects of 1,25(OH)_2_D_3_ and HMB on serum IL-6 levels

**Fig 5:**
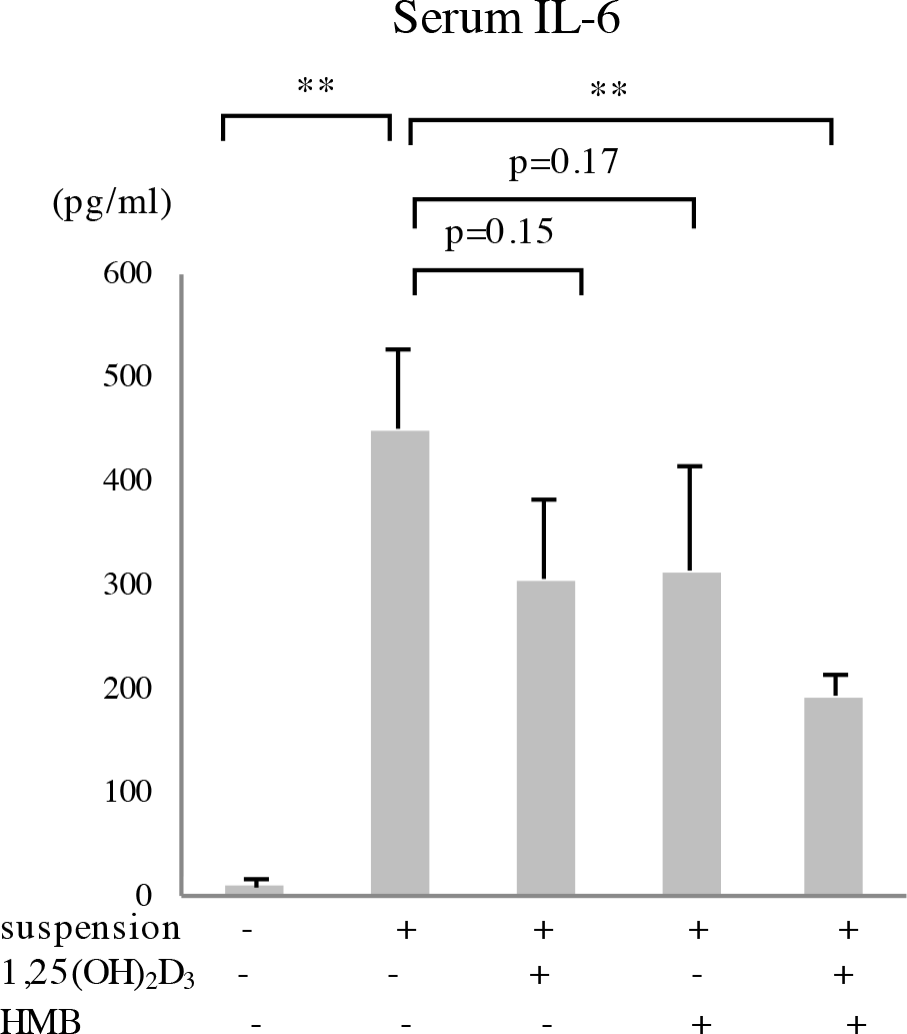
Effects of 1,25(OH)_2_D_3_ and HMB on the tail suspension-induced serum IL-6 surge. Twelve-week-old C57BL/6J male mice were administered 1,25(OH)_2_D_3_, HMB or both by gavage for seven days and then subjected to two weeks of tail suspension. Serum IL-6 level was determined by ELISA. n = 4, **p<0.01.

To examine the effects of 1,25(OH)_2_D_3_ and HMB on the tail suspension-induced serum IL-6 surge, we used another group of mice. This time, the mice were divided into five groups: control, tail suspension, tail suspension+1,25(OH)_2_D_3_, tail suspension+HMB, and tail suspension+1,25(OH)_2_D_3_+HMB (each n = 4). After one week of pretreatment, the mice were subjected to two weeks of tail suspension. Although the difference was not significant, both 1,25(OH)_2_D_3_ and HMB inhibited the IL-6 surge (p = 0.15 and 0.17, respectively). When 1,25(OH)_2_D_3_ and HMB were administered in combination, the IL-6 surge was significantly inhibited (Fig 5).

## Discussion

In the present study, we demonstrated that the serum IL-6 level was elevated in mice subjected to tail suspension and that the inhibition of IL-6R ameliorated the resulting muscle atrophy. The expression of MuRF1 was repressed by the inhibition of IL-6R, but the expression of atrogin-1 was not affected. The novelty of the present study is that systemic IL-6 might be involved in unloading-induced atrophy, and that the inhibition of IL-6 repressed the atrophy. We also showed that both 1,25(OH)_2_D_3_ and HMB inhibited atrogene expression and muscle atrophy in mice.

Several disuse-induced atrophy models have been developed and examined in addition to the tail suspension model. One is the cast immobilization model, in which the unilateral hindlimb is wrapped in casting tape so that the hip, knee, and ankle are flexed. When rats were subjected to this intervention for four weeks, the expression of TNFα and IL-6 was elevated in the hindlimb skin [33]. In the present study, the serum IL-6 level and IL-6 expression in the whole bone marrow were elevated in the tail-suspended mice (Fig 2A and 2B). This finding is consistent with the results of a previous study that reported the induction of IL-6 secretion from bone marrow stromal cells by tail suspension [40]. Some studies have suggested the relation between endotoxin and tail suspension. Gut motility is slower during exposure to microgravity [41]. Vascular changes including increased blood flow to the gastrointestinal tract have been reported after tail suspension [42]. Compared to those in non-suspended mice, the level of LPS in the portal vein and neutrophil count and steatosis in the liver are increased in tail-suspended mice [43]. The portal vein endotoxin levels are approximately 100 pg/mL [43]. It is possible that the intestinal mucosal barrier is damaged in tail-suspended mice, which results in the translocation of bacteria and endotoxins from the gut lumen into the circulation. In the present study, the expression of IL-6 in the liver was not significantly elevated on day 7 after tail suspension (Fig 2B). Thus, although elevated serum LPS levels might have induced IL-6 expression in the bone marrow cells, this may not have been sufficient to increase the IL-6 expression in the liver.

We did not observe elevated IL-6 expression in the soleus muscles or other tissues (Fig 2B). Therefore, it is unlikely that the serum IL-6 was derived from soleus muscles or other atrophying muscles. It is possible that tail suspension increases the load on the forelimbs of the mice and that forelimb skeletal muscles produced IL-6 similar to that observed during exercise. However, the forelimbs are loaded similarly to the control mice during tail suspension [44], and the weight of forelimb muscles is not increased in rats after fourteen days of tail suspension [45]. Taken together, the elevated serum IL-6 is unlikely to have originated from skeletal muscles.

In the present study, inhibition of IL-6 partially ameliorated the muscle atrophy (Fig 3B and 3C). IL-6- transgenic mice exhibit decreased skeletal muscle mass, and an anti-mouse IL-6R antibody completely abrogates atrophy [27]. A previous study showed that IL-6 alters the phosphorylation of ribosomal S6 kinase and STATs (Signal Transducers and Activator of Transcriptions), indicating a shift in the balance of growth factor-related signaling toward a more catabolic profile [26]. Another study suggested that IL-6 and serum amyloid A produced in the liver synergistically increase MuRF1 and atrogin-1 expression by inducing SOCS-3 expression and impairing its downstream insulin/IGF-1 signaling in skeletal muscles [46]. Likewise, the serum IL-6 might alter the phosphorylation of ribosomal S6 kinase and STATs and might even work with amyloid A to affect atrogene expression in tail-suspended mice. MuRF1 and atrogin-1 share several regulatory factors such as FoxO, MAPK and NF-κB [5, 6, 47]. In our study, MuRF1 expression was inhibited by MR16-1, while atrogin-1 expression was not (Fig 3A), indicating that these genes are differentially regulated by IL-6. The effects of MR16-1 on muscle atrophy was partial (Fig 3B and 3C), suggesting that IL-6 is not the only factor involved in the development of disuse-induced muscle atrophy. Further studies are needed to elucidate the molecular mechanism by which IL-6 regulates atrogene expression in skeletal muscles.

We also showed that HMB and 1,25(OH)_2_D_3_ significantly repressed the expression of atrogenes and ameliorated atrophy (Fig 4A–4C). These compounds inhibited the tail suspension-induced serum IL-6 surge on day 14 (Fig 5). When 1,25(OH)_2_D_3_ and HMB were administered in combination, their effect on the serum IL-6 surge was significant. As described in the Introduction section, 1,25(OH)_2_D_3_ and HMB act via distinct mechanisms and thus have additive effects. One possibility is that these compounds repressed the atrogene expression via IL-6R by the inhibition of IL-6 expression. However, when only one of the compounds was administered, the serum IL-6 surge was not significantly inhibited. Thus, it is also possible that atrogene expression and serum IL-6 levels are regulated via independent pathways in response to HMB or 1,25(OH)_2_D_3_.

Few studies have previously shown that vitamin D represses atrogene expression [16]. However, we have demonstrated for the first time that vitamin D represses the expression of MuRF1 and atrogin-1 in a mouse model of disuse-induced atrophy. In one study, HMB administered to tail-suspended rats inhibited myonuclear apoptosis during the recovery from tail suspension-induced muscle atrophy in aged rats [48]. In the present study, pretreatment with HMB prevented muscle atrophy. Our data are consistent with those of previous studies, showing that HMB represses the expression of MuRF1 and atrogin-1 [8–10]. In a previous study, HMB did not repress the expression of MuRF1 and atrogin-1 and did not protect rats from skeletal muscle atrophy induced by monolateral hindlimb immobilization [49]. In that study, immobilization was performed in one hindlimb by applying a cast with total plantar extension, and the knee was fully extended. The amount of HMB administered was 600 mg/kg/day, which is greater than the amount used in the present study. Differences in species and the method of immobilization may have affected the results.

In aged mice, the serum IL-6 level was higher and skeletal muscle mass was lower than those in their younger counterparts (Fig 2C). Disuse-induced muscle atrophy and aging-related atrophy are similar in some aspects but different in others. The plasma TNFα level is high in tail-suspended mice [43]. Some studies have suggested a relationship between inflammatory cytokines (such as IL-6 and TNFα) and sarcopenia in humans [28, 50]. Both MuRF1 and atrogin-1 are upregulated in denervated muscle, immobilized muscle [1], and the skeletal muscles of aged humans and rats [51, 52]. Disuse primarily causes slow fiber atrophy with a slow-to-fast fiber type shift [53], while age-related muscle atrophy is predominant in fast fibers [54]. The administration of MR16-1 in aged mice might provide us additional insights into the mechanisms by which IL-6 affects age-related muscle atrophy.

In summary, the inhibition of IL-6 can suppress atrogene expression and ameliorate tail suspension-induced muscle atrophy. Therefore, IL-6 inhibition may also be therapeutic for other types of muscle atrophy involving atrogenes.
